# Clinicopathological predictors of postoperative upstaging to invasive ductal carcinoma (IDC) in patients preoperatively diagnosed with ductal carcinoma in situ (DCIS): a multi-institutional retrospective cohort study

**DOI:** 10.1007/s12282-021-01225-0

**Published:** 2021-02-18

**Authors:** Kiyo Tanaka, Norikazu Masuda, Naoki Hayashi, Yasuaki Sagara, Fumikata Hara, Takayuki Kadoya, Akira Matsui, Chieko Miyazaki, Tadahiko Shien, Eriko Tokunaga, Takako Hayashi, Naoki Niikura, Shigeto Maeda, Yoshihumi Komoike, Hiroko Bando, Chizuko Kanbayashi, Hiroji Iwata

**Affiliations:** 1 Department of Breast and Endocrine Surgery, Toranomon Hospital, 2-2-2 Toranomon, Minato-ku, Tokyo, 105-8470 Japan; 2 Department of Surgery, Breast Oncology, National Hospital Organization Osaka National Hospital, 2-1-14 Hoenzaka, Chuo-ku, Osaka, 540-0006 Japan; 3 Department of Breast Surgery, St. Luke’s International Hospital, 9-1 Akashicho, Chuo-ku, Tokyo, 104-8560 Japan; 4 Department of Breast Surgical Oncology, Sagara Hospital, 3-31 Matsubaracho, Kagoshima, 892-0833 Japan; 5 Department of Breast Surgery, Shikoku Cancer Center, 160 Kora, Umemotocho, Matsuyama, Ehime 791-0280 Japan; 6 Department of Breast Surgery, Hiroshima University Hospital, 1-2-3 Kasumi, Minami-ku, Hiroshima, 734-8551 Japan; 7 Department of Breast Surgery, National Hospital Organization, Tokyo Medical Center, 2-5-1 Higashigaoka, Meguro-ku, Tokyo, 152-8902 Japan; 8 Department of Breast Surgery, Jichi Medical University Hospital, 3311-1 Yakushiji, Simono, Tochigi, 329-0498 Japan; 9 Department of Breast and Endocrine Surgery, Okayama University Hospital, 2-5-1 Shikatacho, Kita-ku, Okayama, 700-8558 Japan; 10 Department of Breast Oncology, National Hospital Organization Kyusyu Cancer Center, 3-1-1 Nodame, Minami-ku, Fukuoka, 811-1395 Japan; 11 Department of Breast Surgery, National Hospital Organization, Nagoya Medical Center, 4-1-1 Sannomaru, Naka-ku, Nagoya, 460-0001 Japan; 12 Department of Breast Surgery, Tokai University Hospital, 143 Shimokasuya, Isehara, Kanagawa 259-1193 Japan; 13 Department of Surgery, National Hospital Organization Nagasaki Medical Center, 2-1001-1 Kubara, Omura, Nagasaki 856-8562 Japan; 14 Department of Breast Surgery, Kindai University Hospital, 377-2 Onohigashi, Sayama, Osaka, 589-8511 Japan; 15 Department of Breast and Endocrine Surgery, Faculty of Medicine, University of Tsukuba, 1-1-1 Tennodai, Tsukuba, Ibaraki 305-8576 Japan; 16 Department of Breast Oncology, Niigata Cancer Center Hospital, 2-15-3 Kawagishicho, Chuo-ku, Niigata City, Niigata 951-8566 Japan; 17 Department of Breast Oncology, Aichi Cancer Center Hospital, 1-1 Kanokoden, Chikusa-ku, Nagoya, 464-8681 Japan

**Keywords:** Breast cancer, Ductal carcinoma in situ, Dynamic magnetic resonance imaging, Predictive factors, Upstaging

## Abstract

**Background:**

We conducted a prospective study with the intention to omit surgery for patients with ductal carcinoma in situ (DCIS) of the breast. We aimed to identify clinicopathological predictors of postoperative upstaging to invasive ductal carcinoma (IDC) in patients preoperatively diagnosed with DCIS.

**Patients and methods:**

We retrospectively analyzed patients with DCIS diagnosed through biopsy between April 1, 2010 and December 31, 2014, from 16 institutions. Clinical, radiological, and histological variables were collected from medical records.

**Results:**

We identified 2,293 patients diagnosed with DCIS through biopsy, including 1,663 DCIS (72.5%) cases and 630 IDC (27.5%) cases. In multivariate analysis, the presence of a palpable mass (odds ratio [OR] 1.8; 95% confidence interval [CI] 1.2–2.6), mammography findings (≥ category 4; OR 1.8; 95% CI 1.2–2.6), mass formations on ultrasonography (OR 1.8; 95% CI 1.2–2.5), and tumor size on MRI (> 20 mm; OR 1.7; 95% CI 1.2–2.4) were independent predictors of IDC. Among patients with a tumor size on MRI of ≤ 20 mm, the possibility of postoperative upstaging to IDC was 22.1%. Among the 258 patients with non-palpable mass, nuclear grade 1/2, and positive for estrogen receptor, the possibility was 18.1%, even if the upper limit of the tumor size on MRI was raised to ≤ 40 mm.

**Conclusion:**

We identified four independent predictive factors of upstaging to IDC after surgery among patients with DCIS diagnosed by biopsy. The combined use of various predictors of IDC reduces the possibility of postoperative upstaging to IDC, even if the tumor size on MRI is larger than 20 mm.

## Introduction

The increase in breast cancer screening programs has contributed to a dramatic increase in the incidence of ductal carcinoma in situ (DCIS), and more than 20% of breast cancers diagnosed by screening mammography (MMG) are DCIS according to a recent study [1]. It has also been reported that approximately 80% of breast cancers diagnosed by calcifications on screening MMG are DCIS [1].

Surgical management is the current standard approach for DCIS. For breast lesions, breast-conserving surgery followed by radiotherapy or total mastectomy with or without reconstruction is performed. For sentinel lymph nodes, the Japan Breast Cancers Guideline recommends that sentinel lymph node biopsy (SLNB) can be omitted in DCIS patients treated with breast-conserving surgery and predicted to have no invasion [2]; in daily practice, SLNB is sometimes omitted. DCIS has a very good prognosis, and especially for patients with low-risk DCIS, the current standard surgery does not contribute to the improvement of life prognosis [3]. Several randomized controlled trials, such as the COMET [4, 5], LORD [6], and LORIS [7] trials, are currently investigating the feasibility and non-inferiority of active surveillance with or without endocrine therapy for managing low-risk DCIS. In Japan, the single-arm JCOG1505 (LORETTA trial, UMIN 000028298) [8] has begun to confirm non-inferiority of endocrine therapy alone compared to surgery for estrogen receptor-positive, low-risk DCIS.

A problem in omitting surgery is that among patients with preoperatively diagnosed DCIS, 8.3–43.6% presents upstaging to invasive carcinoma as determined by examination of postoperative specimens [9–14]. Furthermore, the frequency of axillary node-positive among patients preoperatively diagnosed with DCIS is 2.5–6.8% [15–17]. Thus, better preoperative information is important to predict DCIS in the final pathological diagnosis so as not to administer overly intensive treatment to patients.

The current study aimed to understand the diagnostic accuracy and treatments of DCIS in institutions with the intention to research individualized DCIS treatment for the future. In addition, we sought to identify clinicopathological predictors of postoperative upstaging to IDC in patients preoperatively diagnosed with DCIS to assist with the provision of adequate surgical procedures.

## Patients and methods

### Patients

We retrospectively reviewed patients diagnosed with DCIS through core needle or vacuum-assisted biopsy between April 1, 2010 and December 31, 2014, from 16 institutions of the Breast Cancer Study Group in Japan Clinical Oncology Group (JCOG). This study was approved by the Institutional Review Boards of each institution. The need for written informed consent was waived due to the retrospective nature of the study, and the patients were provided with a means to opt out.

### Preoperative radiological assessment

All patients routinely underwent clinical examination, MMG, ultrasonography (US), and dynamic magnetic resonance imaging (MRI). The collection items were as follows: presence or absence of a palpable mass as a clinical examination finding, category classifications [18] and the presence or absence of calcification as MMG findings, presence or absence of mass formation, low echoic area and mammary duct ectasia as US findings, tumor size including non-mass enhancement and presence or absence of mass formation as MRI findings. All data were collected from medical records or clinical database by breast oncologists in each institution.

### Pathological assessment

The pre- and postoperative pathological findings, including estrogen receptor (ER), progesterone receptor (PgR), human epithelial growth factor receptor 2 (HER2), DCIS grade (low, intermediate, or high), nuclear grade, and the presence or absence of comedo necrosis, were collected from the pathological reports in each institution. The tumors on both pre- and postoperative specimens were histologically classified using the World Health Organization criteria [19]. ER and PgR were considered positive if reported as a total Allred score of 3–8 or a positive cell occupancy of 1% or more on immunohistochemical analysis. HER2 positivity was defined as a receptor overexpression score of 3 + on immunohistochemical analysis [20]. The Van Nuys classification system was used for DCIS grade, and final postoperative pathological results were classified using the TNM classification.

### Surgical procedure

The breast (partial or total mastectomy) and axillary lymph node (none, SLNB, or axillary lymph node dissection) surgical procedures were collected.

### Adjuvant treatments and follow-up

Adjuvant treatment, including endocrine therapy, radiotherapy, chemotherapy, and additional surgery, were collected from medical records. The recurrence status was also assessed.

### Statistical analysis

Preoperative clinicopathological findings were extracted to determine their association with a postoperative diagnosis upstaging from DCIS to IDC, and logistic regression analysis was used to assess the factors. Variables with a *p* value < 0.0001 in the univariate analysis were included in the multivariate analysis, and a *p* value ≤ 0.05 was considered statistically significant. Statistical analyses were performed using JMP^®^ 12.1 (SAS Institute Inc., Cary, NC, USA).

We considered the relationship between tumor size, including non-mass enhancement on MRI, plus preoperative clinicopathological factors and the possibility of postoperative upstaging to IDC on postoperative specimens. The possibility of postoperative upstaging to IDC was calculated by the number of patients who were preoperatively diagnosed with DCIS as the denominator, and the number of patients postoperatively diagnosed with IDC cancer as the numerator. First, a graph was created by setting the upper limit of the tumor diameter at 5-mm intervals and calculating the ratio of postoperative upstaging to IDC using the dynamic MRI tumor diameter data. Next, in patients with data on dynamic MRI tumor diameter and preoperative clinicopathological factors, the possibility of postoperative upstaging to IDC was calculated in the same manner.

## Results

We identified 2,317 patients diagnosed with DCIS through preoperative biopsies. Among the patients, postoperative diagnosis was special type (mucinous carcinoma) in 2 patients, lobular carcinoma in situ in 5 patients, invasive lobular carcinoma in 2 patients, benign tumor in 8 patients, and no postoperative report in 7 patients. Therefore, excluding these 24 patients, further analysis was performed in a total of 2,293 patients whose final pathological results were DCIS and IDC.

The median age of the 2,293 patients was 52 (interquartile range 17) years old. A total of 1,201 of the 2,293 patients (52.4%) underwent breast-conserving surgery, 1,663 (72.5%) were postoperatively diagnosed with DCIS, and 630 (27.5%) upstaged to IDC (Table 1).

**Table 1:** Patient characteristics

Preoperative clinicopathological diagnosis	Postoperative pathological diagnosis			
	DCIS (*n* = 1663) %		IDC (*n* = 630) %	
Median of age (interquartile range)	51 years old (16)		53 years old (18)	
Findings of palpitation, induration				
+	464	27.9	312	49.5
−	1067	64.2	276	43.8
N/A	132	7.9	42	6.7
Mammography (MMG)				
Classification of MMG				
C1, C2	137	8.2	41	6.5
C3	519	31.2	125	19.8
C4	410	24.7	186	29.5
C5	153	9.2	120	19.0
N/A	444	26.7	158	25.1
Calcification				
+	1229	73.9	475	75.4
−	393	23.6	140	22.2
N/A	41	2.5	15	2.4
Ultrasonography (US)				
Mass formation				
Mass and/or low echoic area	563	33.9	310	49.2
Low echoic area only	760	45.7	250	39.7
N/A	340	20.4	70	11.1
Mammary duct expansion				
+	195	11.7	86	13.7
−	951	57.2	374	59.4
N/A	517	31.1	170	27.0
Dynamic magnetic resonance imaging (MRI)				
Mass formation				
Mass and/or low density area	504	30.3	229	36.3
Low density area only	561	33.7	201	31.9
No abnormality	16	1.0	4	0.6
N/A	582	35.0	196	31.1
Tumor size including non-mass enhancement				
Median (range; mm)	30 mm (4–100 mm)		30 mm (4–100 mm)	
≤ 20 mm	363	21.8	102	16.2
> 20 mm	433	26.0	251	39.8
N/A	867	52.1	277	44.0
Findings of biopsy				
DCIS grade				
Low	305	18.3	108	17.1
Intermediate	382	23.0	144	22.9
High	140	8.4	82	13.0
N/A	836	50.3	296	47.0
Nuclear grade				
1	580	34.9	163	25.9
2	599	36.0	243	38.6
3	160	9.6	91	14.4
N/A	324	19.5	133	21.1
Comedo necrosis				
+	464	27.9	233	37.0
−	839	50.5	309	49.0
N/A	360	21.6	88	14.0
Hormone receptor status				
ER + and/or PgR +	805	48.4	323	51.4
ER − and PgR −	114	6.9	73	11.4
N/A	744	44.7	234	37.1
HER2				
3 +	151	9.1	87	13.8
2 +	39	2.3	12	1.9
0 or 1 +	589	35.4	212	33.7
N/A	884	53.2	319	50.6

In the 630 patients with IDC, the tumor size by T category for pTNM classification was pT1mic (≤ 1 mm) in 136 (21.6%), pT1a (1–5 mm) in 212 (33.7%), pT1b (5–10 mm) in 126 (20.0%), pT1c (10–20 mm) in 80 (12.7%), > 20 mm (> pT2) in 37 (5.7%), and no data in 39 (6.2%).

The axillary operation methods were SLNB in 1,807 patients (78.8%), axillary lymph node dissection in 105 (4.6%), omission in 258 (11.3%), and no data in 123 (5.4%). Ninety-seven patients (4.2%) had lymph node metastasis, including 16 of the 1,663 patients with DCIS (1.0%) and 81 of the 630 patients with IDC (12.9%).

Among the 1,663 patients with DCIS in the final pathological results, 1,403 (84.4%) were hormone receptor-positive in either or both of the pre- or postoperative results, 243 (14.6%) were hormone receptor-negative in both pre- and postoperative pathologically results, and 17 (1.0%) had no data. Moreover, there was variation among institutions in terms of whether patients with hormone receptor-positive DCIS received adjuvant endocrine therapy (median 19.4% [0.0–70.2%]).

A total of 25 (1.5%) of the 1,663 patients with postoperatively diagnosed DCIS had recurrence at the median follow-up period of 33.1 (0–78.6) months, and the most common site of recurrence was the ipsilateral breast (16 of 25 patients).

### Predictive factors of IDC by univariate and multivariate analysis

In the univariate analysis, the following variables were significantly associated with IDC: presence of palpable mass; MMG findings of ≥ category 4; mass formations on US or dynamic MRI; tumor size, including non-mass enhancement on MRI, of > 20 mm; preoperative pathological findings (hormone receptor-negative DCIS, HER2 [3 +], DCIS grade [intermediate or high grade], nuclear grade [2 or 3], and presence of comedo necrosis; Table 2). In the multivariate analysis, the presence of palpable mass (odds ratio [OR] 1.8; 95% confidence interval [CI] 1.2–2.6; *p* = 0.0015), MMG findings (≥ category 4; OR 1.8; 95% CI 1.2–2.6; *p* = 0.0015), mass formations on US (OR 1.8; 95% CI 1.2–2.5; *p* = 0.0019), and tumor size, including non-mass enhancement, on MRI of > 20 mm (OR 1.7; 95% CI 1.2–2.4; *p* = 0.0064) remained as independent predictors of IDC (Table 2). Among 136 patients without all four independent predictors, the possibility of postoperative upstaging to IDC was 10.3% (14/136).

**Table 2:** Predictive factors of invasion by univariate and multivariate analysis

	Univariate				Multivariate		
	OR	RR	(95% CI)	*p* value	OR	(95% CI)	*p* value
The presence of a palpable mass	2.6	2.0	(1.7–2.2)	< 0.0001	1.8	(1.2–2.6)	0.0015
Mammography							
≥ Category 3	1.3	1.2	(0.9–1.6)	0.13			
≥ Category 4	2.1	1.8	(1.5–2.1)	< 0.0001	1.8	(1.2–2.6)	0.0015
The presence of calcification	1.1	1.1	(0.1–1.2)	0.54			
Ultrasonography							
Mass formation	1.9	1.6	(1.4–1.8)	< 0.0001	1.8	(1.2–2.5)	0.0019
Mammary duct ectasia	1.1	1.1	(0.9–1.3)	0.47			
Dynamic magnetic resonance imaging							
Mass formation	1.3	1.2	(1.0–1.4)	0.040			
Tumor size including non-mass enhancement > 20 mm	2.1	1.7	(1.4–2.0)	< 0.0001	1.7	(1.2–2.4)	0.0064
Biopsy findings							
DCIS grade (intermediate or high)	1.2	1.2	(1.0–1.4)	0.16			
DCIS grade (high)	1.6	1.3	(1.0–1.7)	0.033			
≥ NG 2	1.6	1.4	(1.2–1.6)	< 0.0001	1.3	(0.8–2.0)	0.2320
NG 3	1.7	1.4	(1.2–1.7)	0.0006			
The presence of comedo necrosis	1.4	1.2	(1.1–1.4)	0.0037			
Hormone receptor-positive	0.6	0.7	(0.6–0.9)	0.0057			
HER2 [3 +]	1.6	1.4	(1.1–1.7)	0.033			

**Fig. 1:**
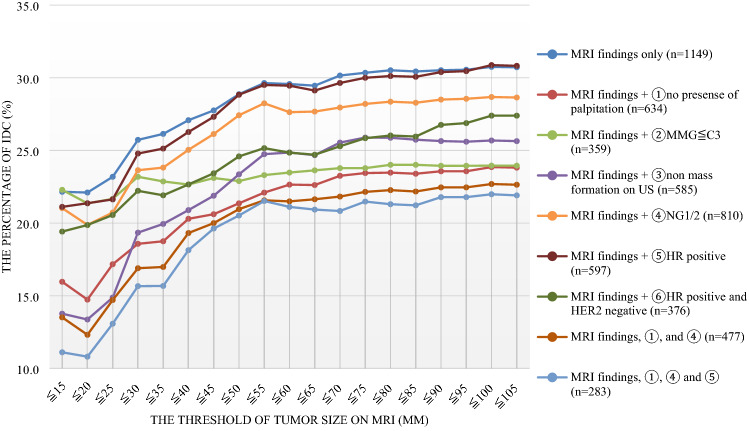
The relationship between tumor size on MRI plus other factors and the percentage of postoperative upstaging to IDC

In total, 1,538 of the 2,317 patients (66.1%) with preoperatively diagnosed DCIS in 16 institutions underwent dynamic MRI (range 26.7–100.0%), and 1,149 patients had detailed dynamic MRI tumor diameter data. Larger threshold of tumor size, including non-mass enhancement, on MRI increases the possibility of postoperative upstaging to IDC (Fig. 1). When a threshold of tumor size, including non-mass enhancement, on MRI was ≥ 50 mm, the possibility of postoperative upstaging to IDC was almost the same. Among patients with a tumor size on MRI of ≤ 20 mm, the possibility of postoperative upstaging to IDC was 22.1%. Among the 258 patients with non-palpable mass, NG1/2, ER-positive DCIS, and detailed tumor diameter data on dynamic MRI, the possibility was 10.8% when the tumor size, including non-mass enhancement, on MRI was ≤ 20 mm. In addition, the possibility was 18.1%, even when the upper limit of the tumor size on MRI was raised to ≤ 40 mm.

## Discussion

In our multi-institutional retrospective study, 630 (27.5%) of the 2,293 patients with preoperatively diagnosed DCIS presented upstaging to IDC on the postoperative specimen. Our results were consistent with those of previous studies (8.3–43.6%) [9–14] and a meta-analysis (25.9%) [13]. Regarding the rate of LN metastasis in patients with preoperatively diagnosed DCIS, our results (4.2%) were consistent with those of previous studies (2.5–6.8%) [15–17]. The small rate of lymph node (LN) metastasis may support omission of upfront SLNB for patients with preoperatively diagnosed DCIS. However, our results showed that 86 (12.9%) of the 630 patients with final pathologically diagnosed IDC had LN metastasis. In recent years, SLNB has been omitted in many cases with preoperative diagnosed DCIS. Since there remains a risk of LN metastasis in patients with IDC, it is important to predict the presence of IDC before surgery to omit SLNB and avoid re-operations of SLNB.

In the univariate analysis, the presence of a palpable mass, MMG findings (≥ category 4), mass formations on US or dynamic MRI, tumor size on MRI (> 20 mm), preoperative pathological findings (hormone receptor-negative, HER2 [3 +], DCIS grade [intermediate or high grade], nuclear grade [2 or 3], and the presence of comedo necrosis) were associated with the presence of IDC in patients who were preoperatively diagnosed with DCIS. These clinicopathological predictors were the same as those described in previous reports [9–17, 21–24]. Some previous studies [13] described that the type of biopsy device (14-gauge automated device versus 11-gauge vacuum) was significantly associated with under-staging. However, we did not collect data on biopsy devices or number of biopsies, although 14- or 16-gauge automated devices (core needle biopsy) are commonly used in daily medical practice.

In previous meta-analyses [13], the tumor size was usually measured by MMG and MRI, or US only if impossible to use MMG. Christiane et al. [25] reported that approximately 40% of DCIS were MRI-only detected lesions, while some later studies [26, 27] have shown the superiority of MRI over MMG for the detection of DCIS (sensitivity of 92% versus 56%, respectively), as well as for the determination of the spread of the disease. On the other hand, one of the weak points of dynamic MRI is overdiagnosis due to background parenchymal enhancement. In daily practice, a dynamic MRI for preoperative assessment in patients with diagnosed DCIS is not routinely performed. Indeed, Roozendaal et al. [28] showed that 409 of 910 patients with preoperatively diagnosed DCIS in four institutions in the Netherlands underwent MRI (average 44.9%; range 5.7–68.2%), while dynamic MRI is more frequently performed in Japan. In the current study, 1,538 of 2,317 patients (66.1%) with preoperatively diagnosed DCIS in 16 institutions underwent MRI (range 26.7–100.0%). We demonstrated a larger threshold of tumor size, including non-mass enhancement on MRI, is associated with an increased possibility of postoperative upstaging to IDC (Fig. 1). The combined use of various IDC predictors to select patients reduces the possibility of postoperative upstaging to IDC, even if the tumor size on MRI is more than 20 mm. Thus, we concluded that dynamic MRI and clinicopathological factors could assist not only with the identification of the extent of resection but also in predicting the possibility of IDC for patients with preoperatively diagnosed DCIS through biopsy to determine the appropriate surgical procedure.

This study has several limitations. First, this is a retrospective study, and there were some missing data of clinical, radiological, and histological variables. However, this study remains one of the largest studies of retrospectively collected data across 16 institutions. Second, the follow-up period was short.

Four active surveillance clinical trials for low-risk DCIS have commenced in the United Kingdom (LORIS), Europe (LORD), United States (COMET), and Japan (JCOG1505, LORETTA trial) [4–8]. These studies are non-inferiority prospective trials to examine the effectiveness and safety of active surveillance compared to surgical based treatment approaches for low-risk DCIS patients, and each of these studies specifies low-risk DCIS with multiple factors. These studies will be important in prospective validation of prognostic factors.

## Conclusion

In conclusion, we identified the following four independent clinicopathological predictive factors of postoperative upstaging to IDC among patients with DCIS diagnosed by biopsy in this retrospective study: presence of a palpable mass, MMG findings (≥ category 4), mass formations on US, and tumor size on MRI (> 20 mm). The combined use of various predictors of IDC reduces the possibility of postoperative upstaging to IDC, even if the tumor size on MRI is larger than20 mm. Thus, we consider that the eligibility criteria of prospective study (JCOG1505) are appropriate. In addition, we could also consider the omission of SLNB among patients with low risk of postoperative upstaging to IDC using the four predictive factors.

## References

[1] Jemal A (2007). Cancer statistics 2007. CA Cancer J Clin.

[2] Komoike Y, Inokuchi M, Itoh T, Kitamura K, Kutomi G, Sakai T (2015). Japan Breast Cancer Society clinical practice guideline for surgical treatment of breast cancer. Breast Cancer.

[3] Sagara Y, Mallory MA, Wong S, Aydogan F, DeSantis S, Barry WT, Golshan M (2015). Survival benefit of breast surgery for low-grade ductal carcinoma in situ: a population-based cohort study. JAMA Surg.

[4] Hwang ES, Hyslop T, Lynch T, Frank E, Pinto D, Basila D (2019). The COMET (Comparison of Operative versus Monitoring and Endocrine Therapy) trial: a phase III randomised controlled clinical trial for low-risk ductal carcinoma in situ (DCIS). BMJ Open.

[5] Youngwirth LM, Boughey JC, Hwang ES (2017). Surgery versus monitoring and endocrine therapy for low-risk DCIS: the COMET trial. Bull Am Coll Surg.

[6] Elshof LE, Tryfonidis K, Slaets L, van Leeuwen-Stok AE, Skinner VP, Dif N (2015). Feasibility of a prospective, randomised, open-label, international multicentre, phase III, non-inferiority trial to assess the safety of active surveillance for low risk ductal carcinoma in situ—the LORD study. Eur J Cancer.

[7] Francis A, Thomas J, Fallowfield L, Wallis M, Bartlett JMS, Brookes C (2014). Addressing overtreatment of screen detected DCIS; the LORIS trial. Eur J Cancer.

[8] Kanbayashi C, Thompson AM, Hwang ES, Partridge AH, Rea DW, Wesseling J. The international collaboration of active surveillance trials for low-risk DCIS (LORIS, LORD, COMET, LORETTA). ASCO 2019, TPS603.

[9] Cserni G, Bianchi S, Vezzosi V, Riccardo A, Rita B, Johannes LP (2007). Sentinel lymph node biopsy in staging small (up to 15mm) breast carcinomas. Results from a European multi-institutional study. Pathol Oncol Res.

[10] Goyal A, Douglas-Jones A, Monypenny I, Sweetland H, Stevens G, Mansel RE (2006). Is there a role of sentinel lymph node biopsy inductal carcinoma in situ? Analysis of 587 cases. Breast Cancer Res Treat.

[11] Tan JC, McCready DR, Easson AM, Leong WL (2007). Role of sentinel lymph node biopsy in ductal carcinoma-in-situ treated by mastectomy. Ann Surg Oncol.

[12] Yen TW, Hunt KK, Ross MI, Mirza NQ, Babiera GV, Meric-Bernstam F (2005). Predictors of invasive breast cancer in patients with an initial diagnosis of ductal carcinoma in situ: a guide to selective use of sentinel lymph node biopsy in management of ductal carcinoma in situ. J Am Coll Surg.

[13] Brennan ME, Turner RM, Ciatto S, Marinovich ML, French JR, Macaskill P (2011). Ductal carcinoma in situ at core-needle biopsy: meta-analysis of underestimation and predictors of invasive breast cancer. Radiology.

[14] Ansari B, Ogston SA, Purdie CA, Adamson DJ, Brown DC, Thompson AM (2008). Meta-analysis of sentinel node biopsy in ductal carcinoma in situ of the breast. Br J Surg.

[15] Chin-Lenn L, Mack LA, Temple W, William C, Robert RQ, Pietro R (2014). Predictors of treatment with mastectomy, use of sentinel lymph node biopsy and upstaging to invasive cancer in patients diagnosed with breast ductal carcinoma in situ (DCIS) on core biopsy. Ann Surg Oncol.

[16] Lee SK, Yang JH, Woo SY, Lee JE, Nam SJ (2013). Nomogram for predicting invasion in patients with a preoperative diagnosis of ductal carcinoma in situ of the breast. Br J Surg.

[17] Osako T, Iwase T, Kimura K, Horii R, Akiyama F (2013). Detection of occult invasion in ductal carcinoma in situ of the breast with sentinel node metastasis. Cancer Sci.

[18] Japan Radiological Society and Japanese Society of Radiological Technology (2014). Mammography guideline.

[19] The World Health Organization (1983). Histological typing of breast tumors. Neoplasma.

[20] Wolff AC, Hammond ME, Schwartz JN, Hagerty KL, Allred DC, Cote RJ (2007). American Society of Clinical Oncology/College of American Pathologists guideline recommendations for human epidermal growth factor receptor 2 testing in breast cancer. J Clin Oncol.

[21] Kondo T, Hayashi N, Ohde S, Suzuki K, Yoshida A, Yagata H (2015). A model to predict upstaging to invasive carcinoma in patients preoperatively diagnosed with ductal carcinoma in situ of the breast: a nomogram to predict DCIS. J Surg Oncol.

[22] Son BK, Bong JG, Park SH, Jeong YJ (2011). Ductal carcinoma in situ and sentinel lymph node biopsy. J Breast Cancer.

[23] Al Nemer AM (2017). Histologic factors predicting invasion in patients with ductal carcinoma in situ (DCIS) in the preoperative core biopsy. Pathol Res Pract.

[24] Park HS, Park S, Cho J, Park JM, Kim Sl, Park B-W (2013). Risk predictors of underestimation and the need for sentinel node biopsy in patients diagnosed with ductal carcinoma in situ by preoperative needle biopsy. J Surg Oncol.

[25] Christiane KK, Simone S, Heribert BB, Eva W, Claudia CL, Roy K (2007). MRI for diagnosis of pure ductal carcinoma in situ: a prospective observation study. Lancet.

[26] Lehman CD, Gatsonis C, Kuhl CK, Hendrick RE, Pisano ED, Hanna L, ACRIN Trial 6667 Investigators Group (2007). MRI evaluation of the contralateral breast in women with recently diagnosed breast cancer. N Engl J Med.

[27] Berg WA, Gutierrez L, NessAiver MS, Carter WB, Bhargavan M, Lewis RS, Ioffe OB (2004). Diagnostic accuracy of mammography, clinical examination, US, and MR imaging in preoperative assessment of breast cancer. Radiology.

[28] van Roozendaal LM, Goorts B, Klinkert M, Keymeulen KBMI, De Vries B, Strobbe LJA (2016). Sentinel lymph node biopsy can be omitted in DCIS patients treated with breast conserving therapy. Breast Cancer Res Treat.

